# Nonlinear Optical Limiting and Radiation Shielding Characteristics of Sm_2_O_3_ Doped Cadmium Sodium Lithium Borate Glasses

**DOI:** 10.3390/ma15062330

**Published:** 2022-03-21

**Authors:** Aljawhara H. Almuqrin, Jagannath Gangareddy, Mahesh M. Hivrekar, A. G. Pramod, M. I. Sayyed, K. Keshavamurthy, Naseem Fatima, K. M. Jadhav

**Affiliations:** 1 Department of Physics, College of Science, Princess Nourah Bint Abdulrahman University, P.O. Box 84428, Riyadh 11671, Saudi Arabia; ahalmoqren@pnu.edu.sa; 2 Department of Post-Graduate Studies and Research in Physics, The National College, Jayanagar, Bengaluru 560070, Karnataka, India; 3 Department of Physics, Dr. Babasaheb Ambedkar Marathwada University, Aurangabad 431004, Maharashtra, India; mmhivre77@gmail.com; 4 Department of Physics, Bangalore University, Bengaluru 560056, Karnataka, India; pramod.virat09@gmail.com; 5 Department of Physics, Faculty of Science, Isra University, Amman P.O. Box 33, Jordan; dr.mabualssayed@gmail.com; 6 Department of Nuclear Medicine Research, Institute for Research and Medical Consultations (IRMC), Imam Abdulrahman Bin Faisal University (IAU), P.O. Box 1982, Dammam 31441, Saudi Arabia; 7 Department of Physics, Vivekananda Institute of Technology, Bengaluru 560074, Karnataka, India; keshav.m85@gmail.com; 8 Department of Physics, Government College, Kalaburagi, Gulbarga 585105, Karnataka, India; naseemfatima030@gmail.com

**Keywords:** nanosecond laser pulses, optical limiting, nonlinear optical, Z-scan, radiation shielding glasses, Sm_2_O_3_

## Abstract

Strong nonlinear absorption (NLA), reduced optical limiting (OL) thresholds, and high radiation shielding parameters are required for the effective use of glasses in the laser radiation and nuclear radiation protecting materials. In view of this, the efficacy of Sm_2_O_3_ on the nonlinear optical (NLO) and OL properties were ascertained (at 532 nm) along with radiation shielding characteristics. The open and closed aperture Z-scan profiles revealed the presence of positive NLA and nonlinear refraction (NLR) phenomena respectively. OL measurements showed the existence of limiting behavior in the studied glasses. The NLA and NLR coefficients were improved while the OL thresholds were decreased as the doping of Sm_2_O_3_ elevated to a higher doping level. These improvements in NLA, NLR coefficients and OL efficiencies were attributed to the non-bridging oxygens and high polarizable Sm^3+^ ions. The NLA and OL results clearly suggest the high (5 mol %) Sm_2_O_3_ doped glass (Sm5CNLB) glass is beneficial to protect the delicate devices and human eye by suppressing the high energy laser light. The theoretical linear attenuation coefficients (LAC) values of the presented Sm*x*CNLB glasses were obtained with the help of Phy-X software between 0.284 and 1.333 MeV. At 0.284 MeV, the maximum values occur and take values between 0.302 (for Sm0CNLB) and 0.409 cm^−1^ (for Sm5CNLB). We found that the LAC for the presented Sm*x*CNLB glasses is a function of Sm_2_O_3_ content, where the LAC tends to increase, corresponding to the high probabilities of interaction, as the content of Sm_2_O_3_ changes from 0 to 5 mol %. The effective atomic number (Z_eff_) for the presented Sm*x*CNLB glasses was examined between 0.284 and 1.333 MeV. As the amount of Sm_2_O_3_ is added, the Z_eff_ increases, and this was observed at any energy.

## 1. Introduction

The investigation on optical limiting (OL) materials, which are able to attenuate transmittance of electromagnetic radiation significantly at strong incident (input) intensities whilst remaining optically transparent for weak intensity situations, has been exaggerated from past few decades owing to their promising ability to protect vulnerable optical components and human organs from harmful high energy radiation in both civilian and military applications, as well as to smooth optical transients [1,2,3]. It has been demonstrated that organic materials are efficient OL materials to use in practical applications. However, the excellent performance occurs normally at the excitation of long pulse durations [1]. In addition, these organic materials possess poor chemical and mechanical stability and strong linear absorption. These predicaments can possibly be overcome in inorganic materials. Out of many inorganic materials, the vitreous inorganic materials are receiving considerable attention and interest in recent decades [1,4,5]. In particular, glasses are believed to be potential materials for these applications [6,7] because glasses exhibit better transparency, a moderate melting point, and thermal stability, along with excellent mechanical and chemical durability [6]. Nonetheless, various phenomena are exploited for optical limiting process viz: nonlinear absorption (NLA) and nonlinear refraction (NLR), nonlinear light scattering (NLS), etc. [1,8,9]. On the other hand, the glasses ideally show NLA such as reverse saturable absorption (RSA) due to absorption of two or three photons simultaneously, which is crucial for activing high OL performance (for real-time OL applications, glasses with large two or three photon absorption are the best candidates) [4]. Therefore, it is necessary and interesting to investigate the glasses for OL applications. Moreover, the fabrication of glass matrices is more convenient in contrast to crystals, with good optical quality and appropriate dimensions. Lastly, the glass materials show instantaneous response to the electromagnetic radiation excitation (ultrafast response times) and high transmission under low intensity [4,5,10]. Various phenomena which are responsible for OL process can be assessed by evaluating the NLO properties of glasses. Thereby, the promising demonstration of glasses for OL applications can be done through the evaluation of NLO properties such NLA, NLR, and NLS, out of which we have ascertained the NLO and NLR features of glasses in the current work for understanding the glasses for OL performances.

According to Yicong Huang et al., optical materials with high NLO coefficients have regained great attention for their potential utilization for the manufacturing of photonic devices in different communication systems, which are based on the optics for the switching, amplification, frequency conversion and phase modulation of optical signals [11]. It has been well-documented that for modulating the NLO characteristics to higher values, a π electron bond system of ions in the materials is more helpful [12]. It has been further revealed that the inorganic materials which contain delocalized π electron clouds in the structural units possess high optical nonlinearities [13]. Taking this into account, in comparison with other inorganic glass composition, borate glass composition achieved motivating interest since it has been observed with the majority consisting of the (BO_3_)^3−^ and (B_3_O_6_)^3−^ (three BO_3_ units) units in the structure. These groups comprise the delocalized π electron cloud, due which it is possible to achieve enhancement in the optical nonlinearities [14]. Furthermore, in recent years the incorporation of “rare earth ions” (REI) in the parent glass composition and correlating the featured optical nonlinearity of glass hosts to higher values has been established [15,16,17]. Kirti Nanda et al. [16] have studied the effect of Sm^3+^ ions on NLO and vibrational spectroscopic features of borate-based glasses. They have elucidated that NLO features are improved with Sm_2_O_3_ content due to increase of polarizability of glass as a whole, owing to the formation of non-bridging oxygens (NBOs) on incorporating the highly polarizable Sm^3+^ ions to the borate glass composition. They have also highlighted using vibrational spectroscopy for the formation of NBOs in the glass structure on incorporation of Sm^3+^ to the glass composition. The reason for selecting this route (doping of REI to tune the NLO characteristics) is due to the fact that the REI are highly polarizable, due to which the REI causes high depolymerization in the structure [16]. Since the NLO susceptibility (χ) is related to the depolymerization of structure, the improvement can be expected. In particular, it was explained that the NLR enhancement is related to the formation of the elongated bonds in the structure [18] and resonance effects [19]. It has been also explained that the enhancement in NLA is related to the improvement in polarizability of the glasses [20,21]. Interestingly, the figure of merit (FOM) expression mentioned in the ref [18] clearly revealed the FOM should be greater than unity for utilizing the glasses for OL applications. This is possible by improving the NLA and controlling the NLR features of the glasses, or in other words, significant enhancement in NLA is more essential than NLR for effective utilization of glassy hosts in OL functionalities. To this, the doping of REI to the host glasses is one of the best methods out of many routes followed by many researchers to tune the NLO results of the glasses [4,5,22,23].

On the other hand, the optical materials such as glasses are widely used in an important field, namely, to prepare radiation shielding materials. These materials are useful since they protect the humans as well as the equipment in the nuclear facilities from the harmful effects of the radiations, especially those that have high energy. There are a number of advantages that encouraged researchers and workers in the field of radiation shielding to use different glass systems as a radiation-protective material. These advantages include the transparency of the glass, and this allows the development of shield glasses that are used to protect the face and eyes during the radiological treatment. Additionally, glasses can be prepared by different simple methods and with low cost, which makes glass a good candidate as cheap radiation shielding material. Several glass systems were recently developed and the radiation attenuation factors were determined; the results from different studies showed that the glasses that contain heavy metal oxides have outstanding shielding performance [24,25,26,27,28]. Moreover, the radiation shielding glasses can be used in dentistry applications [29,30].

In this work, we prepared the glasses with a composition of *x*Sm_2_O_3_–7.5CdO–7.5Na_2_O–15Li_2_O–(70–*x*)B_2_O_3_ and investigated the efficacy of Sm_2_O_3_ on the nonlinear optical (NLO) and OL properties (at 532 nm) along with radiation shielding characteristics.

## 2. Experimental Procedures

The precursor inorganic glasses samples loaded with different concentration of Sm_2_O_3_ were prepared by melt quenching process. The glasses were produced with composition (mol %) *x*Sm_2_O_3_–7.5CdO–7.5Na_2_O–15Li_2_O–(70–*x*)B_2_O_3_, where *x* = 0, 0.1, 0.3, 0.5, 1, 2, 3, 4, and 5 mol %. The chemicals such as Sm_2_O_3_, CdO, Na_2_CO_3_, Li_2_CO_3_ and H_3_BO_3_ (maintained purity of 99.99%) were utilized for the fabrication of glass samples. The homogeneous mixtures of chemicals for 15 g of each batch were filled in a porcelain crucible and kept in a furnace for melting. All the glasses were quenched at 1050 °C, before quenching the melts were remained at that temperature for 1 h. The quenched samples were sent to the annealing, at 400 °C for 4 h. This procedure is important to minimize the internal stress and let the glasses become less fragile. The last step refers to the cutting and polishing of the glass samples to required dimensions. Before sending the glasses to the characterization, the samples were coded as Sm*x*CNLB*;* here *x* represents the amount of Sm_2_O_3_ in the composition. The density values of the samples were measured by following Archimedes principle [31]. The structure features of powdered glass samples were analyzed by recording the XRD from Rigaku X-ray diffractometer (using Cu–Kα radiation source) and Fourier transform infrared (FTIR) spectra from Bruker model vertex–70 FTIR spectrometer (M/s Bruker, Billerica, MA, USA). The NLO and OL measurements on the samples were performed using the Z-scan technique [32]. Owing to simplicity and accuracy, the single beam Z-scan method can be employed for the evaluation of NLO, NLR, and OL properties [32]. NLA and OL characteristics were ascertained by performing the Z-scan measurements In open aperture (OA) mode, while NLR characteristics were ascertained by closed aperture (CA) configuration. In the OA mode, the aperture placed after the sample before the detector was kept fully open and the transmitted light from the sample was completely allowed to enter the detector. Meanwhile, in CA mode, the intensity of the transmitted light from the sample was controlled by adjusting the aperture dimension (S). Therefore, OA can be considered as normalized transmittance where S = 1 while the value of S ranges between 0.1 < S < 0.5 for CA. The Z-scan measurements were done at 532 nm utilizing a frequency-doubled Nd:YAG nanosecond laser (Quanta Ray, M/s Spectra Physics, Mountain View, CA, USA) with 5 nanosecond (ns) pulse width and 1 Hz repetition rate. The specifications involved in the Z-scan experiment are as follows: beam waist was (ω_0_) 15.35 μm, the Rayleigh length (Z_0_) was estimated to be 4.26 mm, focal length of the lens was 3.5 cm, spot-size diameter near the aperture was found to be 15 mm, and effective path length (L_eff_) was found to be 0.92 mm. The obtained data were processed using the procedure explained by Sheik Bahae et al. [32] to evaluate the NLO coefficients.

## 3. Results and Discussion

### 3.1. Structural Properties of SmxCNLB Glasses

**Figure 1:**
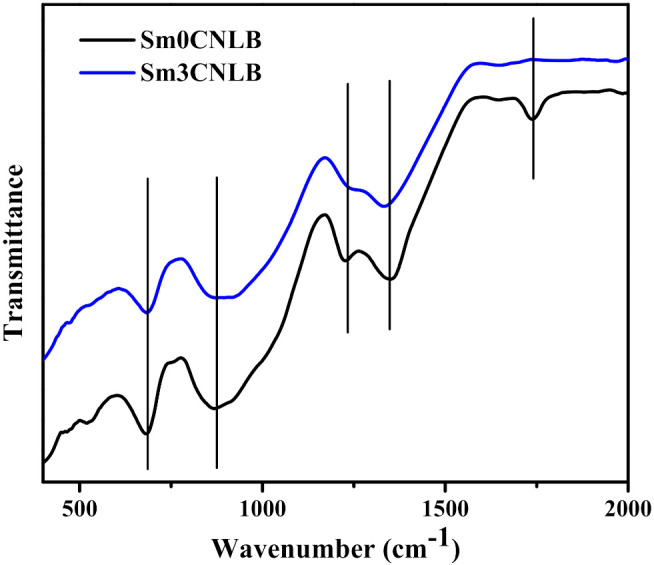
FTIR spectral outcomes of Sm0CNLB and Sm3CNLB glass specimens.

The FTIR spectral outcomes of Sm0CNLB and Sm3CNLB glasses are displayed in Figure 1. There are seven IR bands centered at 470, 681, 873, 1231, 1349, 1643, and 1738 cm^−1^ wavenumbers in the spectra. These bands occurred due to different functional groups of oxide glass composition. The IR band at 470 cm^−1^ of the glass samples is owing to the O–B–O bond bending vibrations. The IR band at 681 cm^−1^ wavenumber is caused due to bending vibration between boron and oxygen (B–O) bands observed in BO_3_ groups [33,34]. The IR band at 873 cm^−1^ belongs to the B–O stretching vibration of BO_4_ units from diborate groups of borate glass. The peaks around 1249 and 1339 cm^−1^ belong to asymmetric B–O vibrational stretch of (BO_3_)^3−^ units from pyro and ortho borate groups in the quaternary undoped borate glass network [35]. The keen observation of FTIR spectra reveal that the bands between 800–1350 cm^−1^ are slightly shifted to low frequency region in Sm_2_O_3_ doped glass compared to base glass. It was demonstrated that the bands around 730–1300 cm^−1^ shifted to lower frequency region instantiate the increase of NBOs and elongation of B–O bonds in BO_4_ structural units [20]. Meanwhile, the opposite nature indicates the decrease of NBOs and shortening of B–O bond lengths in BO_4_ groups [36,37]. Therefore, the change of 800–1350 cm^−1^ band position to slightly lower frequency in Sm3CNLB glass contrast to Sm0CNLB glass is attributed to an increase of NBOs and elongation of B–O bonds in BO_4_ units when high Sm_2_O_3_ is incorporated to the composition [20]. Crystal water in the H–O–H bending mode is credited with the peaks ranging from 1643 to 1738 cm^−1^.

### 3.2. NLO and OL Characteristics of SmxCNLB Glasses

**Figure 2:**
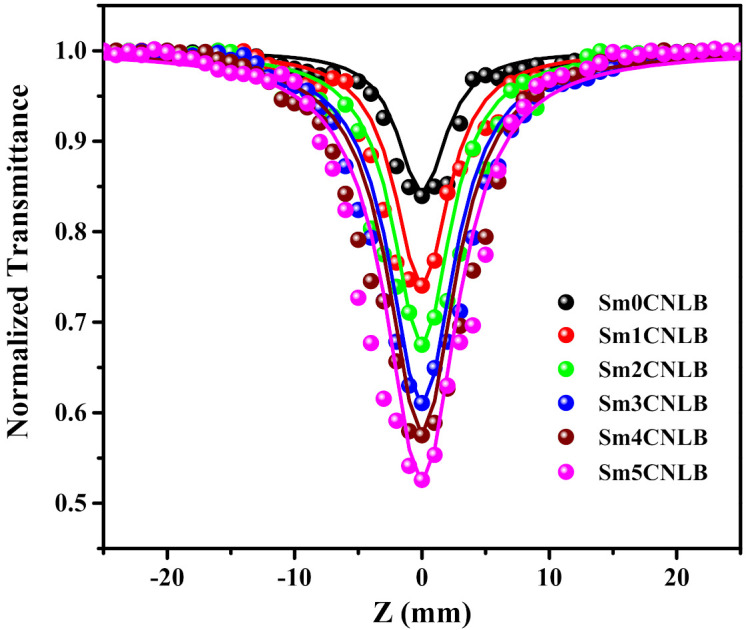
OA Z-scan outcomes of studied Sm*x*CNLB glasses, where *x* = 0, 1, 2, 3, 4 and 5 mol %. The symbols and solid lines indicate the experimental and theoretical OA Z-scan data, respectively.

The OA Z-scan outcomes of studied glasses are presented in Figure 2. The OA Z-scan results of Sm0.1CNLB, Sm0.3CNLBSm, Sm0.5CNLB glass samples are almost similar to that of Sm0CNLB glass (no significant variation has been observed), therefore the OA Z-scan results of these glasses are not displayed in the Figure 2 to avoid messiness in the spectra. However, the values resulted (discussed in the later part of the manuscript) from the fittings are mentioned in the variation graph.

The data in Figure 2 depicted the minimum of normalized transmittance at Z = 0 (focal point). This indicates the nonlinearity for ‘reverse saturable absorption’ (RSA) occurred in investigated glasses [38]. Quantification of the number of photons absorbed for the noticed RSA nonlinear phenomena can be done by fitting the experimental data (symbols in Figure 2) with hypothetical mathematical equation [39]

**Figure 3:**
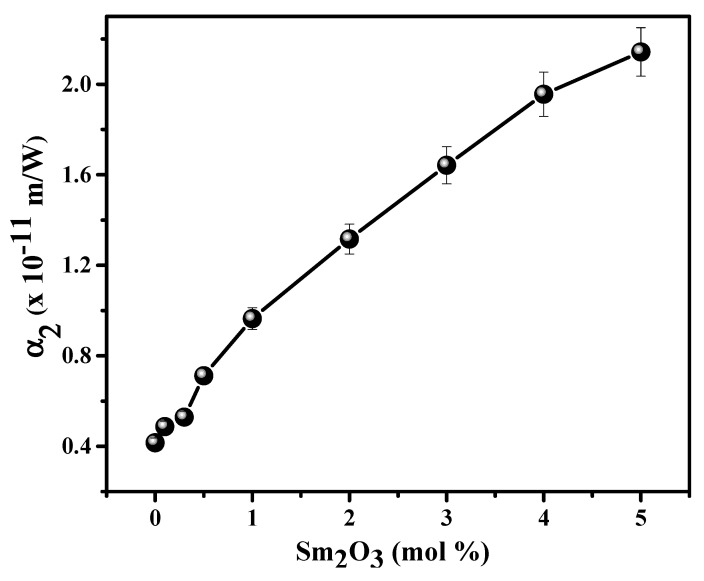
Variation of *α*_2_ with respect to Sm_2_O_3_ content in the glass composition.

(1) TOA(nPA)=11+n−1αnLeffI00/1+Z/Z02n−11n−1

where, *α_n_* is the multi-photon absorption factor (for instance *n* = 2 when the two-photon absorption process and etc.). The symbols in the Equation (1) have the same meanings found in reference [39]. The experimental OA Z-scan data of all the glass matrices well corroborated with theoretical two photon absorption data (solid lines in Figure 2). This unveils the existence of two-photon absorption (2PA) NLO property in the glasses used in the current upon irradiated with high energy radiation. The resulted 2PA coefficients (*α*_2_) of studied glass hosts are provided in Figure 3. In the Figure 3, it is noticeable that the *α*_2_ is almost constant up to 0.5 mol % of Sm_2_O_3_ by considering the fitting errors. The clearly noticeable variation in *α*_2_ has been observed after 1 mol % of Sm_2_O_3_ loading in the composition. The data in Figure 3 also reflects the *α*_2_ increases with the loading of Sm_2_O_3_ content in the composition.

**Figure 4:**
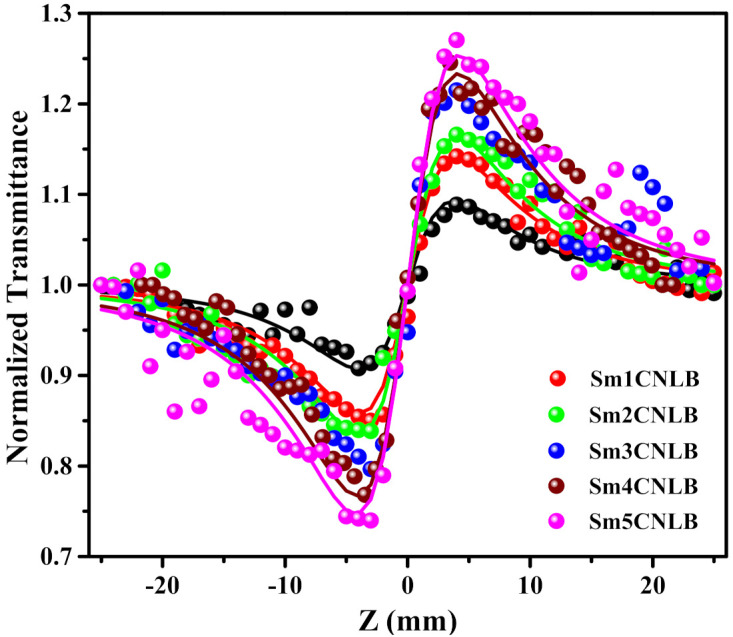
CA Z-scan profiles of studied Sm*x*CNLB glasses, where *x* = 0, 1, 2, 3, 4, and 5 mol %. The symbols and thick lines indicate the experimental and theoretical CA Z-scan data respectively.

The CA Z-scan spectra of studied glass samples are showed in Figure 4. Here also are the CA Z-scan results of Sm0.1CNLB, Sm0.3CNLBSm. Sm0.5CNLB glass samples are not displayed due to similarity of the data with Sm0CNLB glass with the intention of avoiding messiness in the spectra.

**Figure 5:**
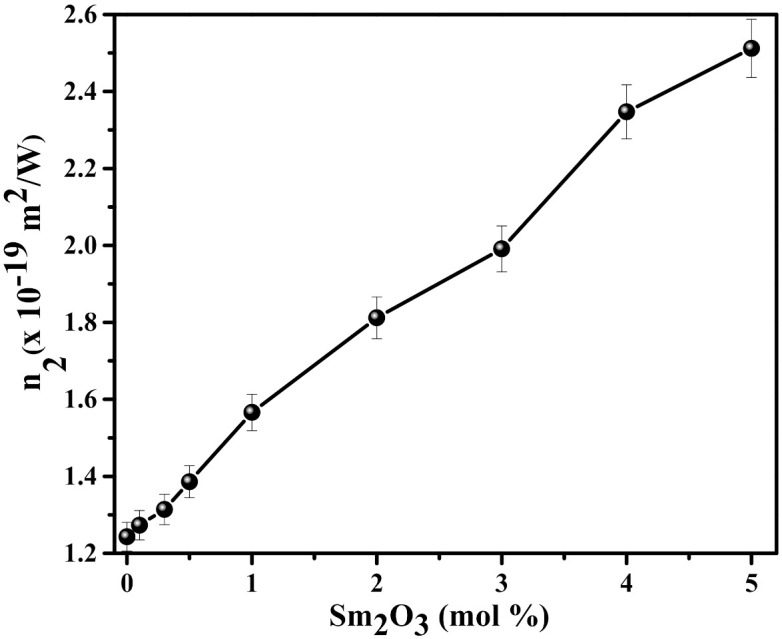
Variation of n2 as the function of Sm_2_O_3_ content in the glass composition.

The data in Figure 4 depicts the pre-focal minima and post-focal maxima of normalized transmittance. This is the signature for positive nonlinear refraction (*n*_2_ > 0) occurred in investigated glass matrices. This positive nonlinear refraction is manifested to ‘self-focusing’ phenomena that occurs upon irradiating with the high fluence laser light [40]. Usually, the *n*_2_ resulted due to phase mismatch (ΔΦ_0_) which was estimated by fitting experimental CA Z-scan data (symbols in Figure 4) using the data obtained from the following equation (solid lines in the Figure 4) [39].

(2) $$T_{CA}=1+\frac{4\Delta \varphi_{0}\left(Z/Z_{0}\right)}{\left[1+{\left(Z/Z_{0}\right)}^{2}\right]\times \left[9+{\left(Z/Z_{0}\right)}^{2}\right]}$$

using ΔΦ_0_ values the nonlinear refractive indices (*n*_2_) were calculated through the below formula [39]

(3) $$n_{2}\left(m^{2}W^{-1}\right)=\frac{\left|\Delta \varphi_{0}\right|\lambda }{2\pi I_{00}L_{eff}}$$

the notations in Equations (2) and (3) owing same meanings found in the reference [37]. The computed *n*_2_ magnitudes for all the glasses are plotted in Figure 5 with respect to Sm_2_O_3_ concentration. Taking into account the experimental errors, it is evident that the *n*_2_ values are almost constant up to 0.5 mol % of Sm_2_O_3._ Improvement in the *n*_2_ values were also observed with the Sm_2_O_3_ content in the composition.

The NBOs and polarizabilities of RE ions cause the magnification of NLO features in the present glass matrices. The enhancement in *α*_2_ and *n*_2_ (1 mol % onwards) with Sm_2_O_3_ concentration is related to these NBOs generated when Sm_2_O_3_ is substituted for B_2_O_3_ in the matrix and polarizabilities of Sm^3+^ ions. The density of the glasses measured by Archimedes showed an increasing trend from 2.642 to 2.997 gcm^−3^ with incorporation of Sm_2_O_3_ from 0 to 5 mol % in the composition. Additionally, values of molar volume were found to be an increasing trend from 25.47 to 27.21 cm^3^mol^−1^ when Sm_2_O_3_ was loaded from 0 to 5 mol % for B_2_O_3_ in the present glass composition. The increasing trend of density with Sm_2_O_3_ is an expected outcome since the heavier Sm_2_O_3_ (348.72 a.m.u) substituted for relatively for lighter B_2_O_3_ (69.62 a.m.u) in the glass matrix. This results in an increase of the density values of the glass as the Sm_2_O_3_ doping elevated to higher level. Increase of molar volume of the glass as whole with Sm_2_O_3_ content is attributed to the modifier role of Sm^3+^ [16]. These attenuations such as an increase in density and an increase in molar volume of the glass as a whole with Sm_2_O_3_ content are observed because of the glass network fragmentation between cations (i.e., B^3+^) and oxygen anions, which results in the generation of NBOs in the structure when the Sm_2_O_3_ is loaded for B_2_O_3_ as the modifier [39]. The NBOs are weakly coordinated to network cations, whose valence electrons produce large charge removals because of the high electric field generated due to the high energy laser radiation used for irradiation. These large charge displacements produced by the valence electrons of NBOs results in the improvement in NLA (i.e., *α*_2_) and NLR (i.e., *n*_2_) glass matrices with increase of Sm_2_O_3_ concentration [20,41]. However, the generated NBOs content might be very less in the lower concentration of Sm_2_O_3_ (i.e., up to 0.5 mol %) and this less NBOs content may not be sensible for the optical nonlinearity thereby the NLA (i.e., *α*_2_) and NLR (i.e., *n*_2_) results are almost constant up to 0.5 mol % of Sm_2_O_3_. Further, when the high polarizable Sm^3+^ ions (1.16 Å^3^) replaced the B^3+^ ions (0.002 Å^3^) weakly polarizable [42] in the glass composition. This is the source of intensification of polarizability in the present glass composition, thereby the polarizability values increase with the Sm_2_O_3_ content raised to higher doping concentration. For validating this inference, the molar electronic polarizability (*α_e_*) of the present glass composition has been calculated by using the equation found in reference [20]. The computed *α_e_* values increases from 3.13 to 4.20 Å^3^ when the Sm_2_O_3_ loaded from 0 to 5 mol %. Further, Nanda et al. [16] have studied the effect of Sm^3+^ ions on optical absorption and NLO features of borate-based glasses. They have not observed any absorption peaks near 532 nm (at which the NLO features are evaluated). Therefore, the resonance and/or energy transfer mechanisms are ruled out for the improved NLO features with Sm_2_O_3_ content. Which means the enhancement in NLO features are ascribed to NBOs formed in the structure of glass and high polarizabilities of glasses occurred on incorporation of Sm^3+^ ions to the glass system. The NLO results of certain REI doped glasses are furnished in Table 1 for the sake of comparison [16,17,39,40]. On comparing the values found in Table 1 with the highest *α*_2_ and *n*_2_ values obtained in present work, it is identified that the *α*_2_ values are compatible with other borate-based glasses doped with Sm^3+^, Er^3+^ and Eu^3+^ ions. However, the *n*_2_ values are one order lesser in magnitude for the present glasses. Since the significant enhancement in NLA is essential than NLR to utilize glassy hosts in OL functionalities. To this, it can be mentioned that high Sm^3+^ doped glasses used in the present work are competing materials for OL applications in comparison with other RE doped glasses.

**Figure 6:**
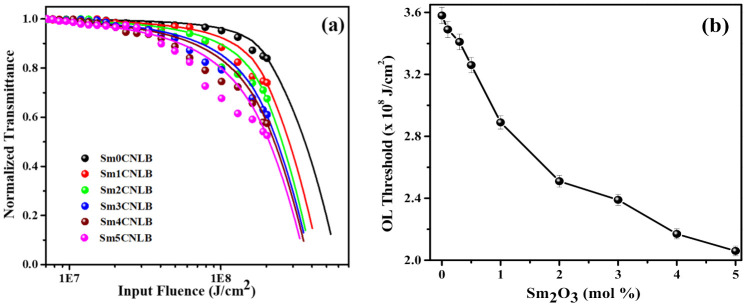
(**a**) OL patterns of examined Sm*x*CNLB glasses, where *x* = 0, 1, 2, 3, 4, and 5 mol %. (**b**) Variation of OL thresholds with Sm_2_O_3_ content in the glass composition. The solid symbols and solid lines in (**a**) represent the measured and theoretical OL data respectively.

Basically, the OL hosts are crucial for the fabrication of laser protection devices to suppress the high energy laser light and protect the sensitive devices. To gain more information about the NLO properties, the OL features of the studied glasses have been investigated. In the experiment, the nonlinear transmission of glass specimens was recorded as the function of the input fluence, and results are presented in Figure 6a. OL property of the materials is sensitive to the incident energy of the laser light [43]. The usefulness of OL materials can be analyzed using the OL threshold, which can be defined as the input energy of laser light at which the normalized transmittance falls to 0.5 (50% of the input fluence). The OL process takes place when the fluence of input laser light goes beyond this OL threshold, at which the material possesses strong NLA and NLR, thereby resulting in a decrease of transmittance [39]. The obtained OL threshold values are presented in the Figure 6b in the form variation with respect to Sm_2_O_3_ content. The values in Figure 6b clearly unveil that the OL performance is increases with Sm_2_O_3_ content (OL threshold values are decreasing with Sm_2_O_3_) which clearly suggest the OL performance is superior for 5 mol % of Sm_2_O_3_ doping. Generally, optical limiter should be manufactured with the materials having high values of *α*_2_ and low OL threshold factors [2]. The data in Figure 3 and Figure 6b revealing the *α*_2_ is improving with Sm_2_O_3_ content and OL threshold is decreasing for Sm_2_O_3_ variation in the composition. Therefore, the high (5 mol %) Sm_2_O_3_ containing glass (Sm5CNLB) is beneficial for fabricating the laser protection devices. It has been reported that the NBOs and Sm^3+^ ions are considered as the main contributors for dominating the NLO coefficients of borate glasses owing their valence electrons and high polarizabilities respectively [16]. Therefore, these NBOs and Sm^3+^ ions are favouring the enhancement of NLA (RSA, i.e., 2PA) through strong light-matter interaction, resulting in the notable increase in the OL efficiency.

### 3.3. Radiation Shielding Characteristics

**Figure 7:**
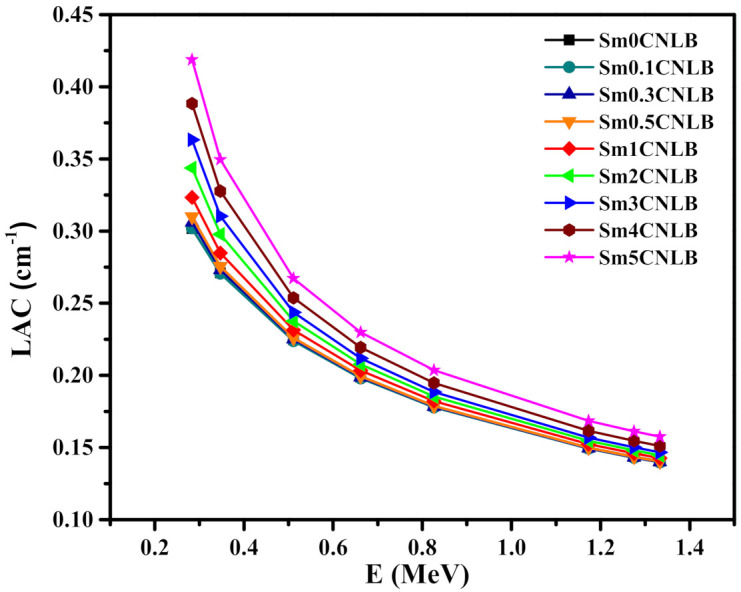
The theoretical linear attenuation coefficient values of the presented Sm*x*CNLB glasses.

The theoretical LAC values of the presented Sm*x*CNLB glasses were obtained with the help of Phy-X software between 0.284 and 1.333 MeV [44]. The values of LAC are plotted in Figure 7. It is important to state that at such energy range, the Photoelectric effect and the Compton scattering are the main interaction processes between the photons and the atoms of the Sm*x*CNLB samples.

This will help us to understand the LAC trend observed in Figure 7. In each glass, the LAC decreases as the energy goes up from 0.284 to 1.333 MeV. This result implies that high interaction chances between photons and electrons in the presented Sm*x*CNLB glasses with low energy of gamma radiation. At 0.284 MeV, the maximum values occur and take values between 0.302 (for Sm0CNLB) and 0.409 cm^−1^ (for Sm5CNLB). The influence of Sm_2_O_3_ content on LAC values was also noticed. This suggests that the LAC for the presented Sm*x*CNLB glasses is a function of Sm_2_O_3_ content. More precisely, the LAC tends to increase, corresponding to the high probabilities of interaction, as the content of Sm_2_O_3_ changes from 0 to 5 mol %. In our glasses, the content of CdO, Na_2_O, and Li_2_O are fixed, thus the replacement of B_2_O_3_ by Sm_2_O_3_ in the Sm*x*CNLB samples increases the number of electrons that can interact with the photons, since the interaction probability of Sm (with atomic number of 62) is much higher than B (with an atomic number of 5). If we look to the LAC for the samples with 0 and 0.1 mol % of Sm_2_O_3_ and the two samples with 0 and 5 mol % of Sm_2_O_3_, we can see the influence of the Sm_2_O_3_ content on the LAC. For Sm0CNLB and Sm0.1CNLB samples, the LAC slightly changes from 0.3017 to 0.3019 cm^−1^ at 0.284 MeV, while it is notably changes from 0.3017 to 0.4187 cm^−1^ due to the increment of Sm_2_O_3_ from 0 to 5 mol %.

**Figure 8:**
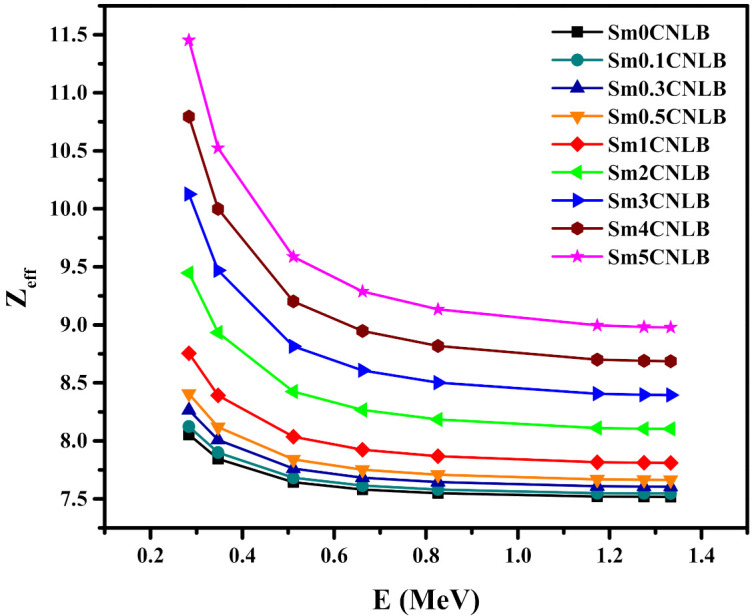
The effective atomic number values of the presented Sm–CNLB glasses.

We evaluated the effective atomic number (Z_eff_) for the presented Sm–CNLB glasses and examined the influence of the amount of Sm_2_O_3_ used in the glasses on the Z_eff_ (see Figure 8). Apparently, the Z_eff_ increases in the order of Sm0CNLB to Sm5CNLB. As the amount of Sm_2_O_3_ is added, the Z_eff_ increases, and this was observed at any energy. In the prepared glasses, the concentrations of CdO, Na_2_O, and Li_2_O were fixed, while the Sm_2_O_3_ is increased at the expense of B_2_O_3_, and since the atomic number of Sm is higher than that of B, the replacement of B_2_O_3_ by Sm_2_O_3_ causes an increase in the electron number per unit atom in the sample, so we found an enhancement in the Z_eff_ values as we move from Sm0CNLB to Sm5CNLB. At 0.284 MeV, the Z_eff_ is 8.05 for the free Sm_2_O_3_ sample, and this increases to 11.45 for the sample with 5 mol % of Sm_2_O_3._ At 0.347 MeV, the Z_eff_ values for the glasses with 0 and 5 mol % of Sm_2_O_3_ is 7.84 and 10.52.

If we look at the Z_eff_ values at specific composition, we see that the Z_eff_ decreases with increasing the energy. This result implies that the Sm*x*CNLB glasses have good attenuation competence when applying in a low energy radiation application.

**Figure 9:**
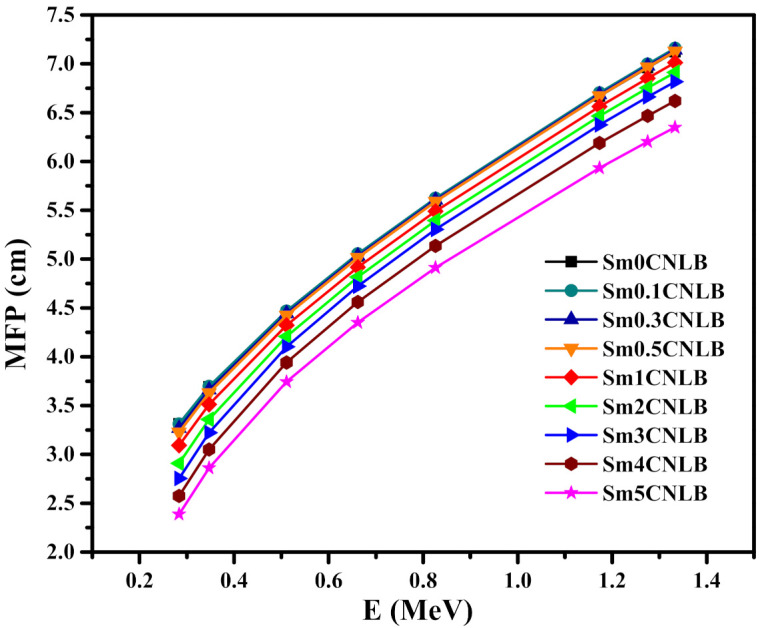
The mean free path (MFP) of the Sm*x*CNLB glasses.

The mean free path (MFP) of the Sm*x*CNLB glasses is illustrated against increasing photon energy in Figure 9.

**Figure 10:**
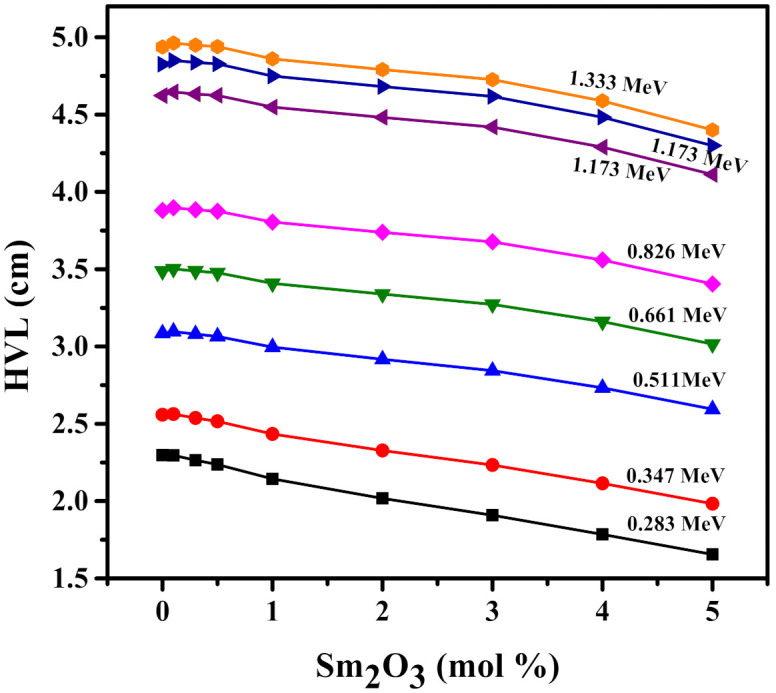
The relation between the half value layer and the Sm_2_O_3_ content in the Sm*x*CNLB glasses.

The trends of the MFP figure as similar to the MFP trends observed for other glass systems [45]. First, MFP increases with energy. For the Sm0CNLB glass, its MFP increases from 3.314 cm at 0.284 MeV to 7.123 cm at 1.333 MeV, while the MFP of the Sm5CNLB is equal to 2.388 and 6.349 cm at the same respective energies. This trend also occurs because the atoms within the materials have a harder time absorbing the incoming photons at higher energies, increasing the distance between subsequent collisions, or MFP. Furthermore, the sample with the least Sm_2_O_3_ content, Sm0CNLB, has the greatest MFP of all energies, while the samples with the greatest Sm_2_O_3_ content, Sm5CNLB, has the least MFP at all energies. This once again indicates that increasing the Sm_2_O_3_ content in the glass system enhances its radiation shielding characteristics. In order to confirm the inverse relation between the radiation shielding attenuation performance and the Sm_2_O_3_ content, we plotted Figure 10, which confirms the inverse dependence on the half value layer (HVL) on the amount of Sm_2_O_3._ This is more notable at lower energies, since the HVL decreases quickly with adding more amount of Sm_2_O_3_. At higher energies, the inverse relation still valid, but the rate of the reduction in HVL is slower.

## 4. Conclusions

In summary, different Sm_2_O_3_ containing glasses were successfully carried out by melt quench technique. NLO, OL, and radiation shielding characteristics were measured and analyzed to understand the effectiveness of Sm_2_O_3_ doping. The OA and CA Z-scan profiles revealed the existence of positive NLA and NLR in the studied glasses. The OL attributes revealed the existence of limiting behavior in the studied glasses. The NLO features were not sensitive up to 0.5 mol % of Sm_2_O_3_ content, the results showed enhancement trend after 1 mol % of Sm_2_O_3_ doping. The NLO unveiled the NLA and NLR factors were improved while the OL profiles revealed the OL thresholds were decreased as the doping of Sm_2_O_3_ raised to higher doping level. These improvements in NLA, NLR, and OL efficiencies were attributed to the NBOs and high polarizable Sm^3+^ ions. Comparison of the NLO results of presently investigated glasses with other REI doped borate-based glasses available in literature reveal the highest Sm^3+^ doped glasses used in the current study are competing materials for OL functionalities suppressing the high energy laser light. Moreover, we evaluated the radiation attenuation factors and examined the influence of the energy and the Sm_2_O_3_ content on the LAC and Z_eff_. The replacement of B_2_O_3_ by Sm_2_O_3_ causes an increase in the electron number per unit atom in the sample, so we found an enhancement in the Z_eff_ values as we move from Sm0CNLB to Sm5CNLB. For the Sm0CNLB glass, its MFP increases from 3.314 cm at 0.284 MeV to 7.123 cm at 1.333 MeV, while the MFP of the Sm5CNLB is equal to 2.388 and 6.349 cm at the same respective energies.

## Figures and Tables

**Table 1:** NLO coefficient of some RE incorporated glasses reported recently for the comparison purpose.

Glass Compositions	Excitation Wavelength and Other Details	*α*_2_ (×10^−11^ m/W)	*n*_2_ (×10^−18^ m^2^/W)	Reference
*x*Sm_2_O_3_–(100–*x*)[0.1BaO–0.4ZnO–0.5B_2_O_3_], *x* = 0 mol % *x*Sm_2_O_3_–(100–*x*)[0.1BaO–0.4ZnO–0.5B_2_O_3_], *x* = 0.5 mol % *x*Sm_2_O_3_–(100–*x*)[0.1BaO–0.4ZnO–0.5B_2_O_3_], *x* = 1.0 mol % *x*Sm_2_O_3_–(100–*x*)[0.1BaO–0.4ZnO–0.5B_2_O_3_], *x* = 1.5 mol % *x*Sm_2_O_3_–(100–*x*)[0.1BaO–0.4ZnO–0.5B_2_O_3_], *x* = 2 mol %	532 nm (5 ns 10 Hz)	0.572 1.128 1.507 1.928 2.746	0.108 0.771 0.899 1.022 1.299	[17]
*x*Er_2_O_3_–(100–*x*)[0.1BaO–0.4ZnO–0.5B_2_O_3_] with *x* = 0.5 mol % *x*Er_2_O_3_–(100–*x*)[0.1BaO–0.4ZnO–0.5B_2_O_3_] with *x* = 1 mol % *x*Er_2_O_3_–(100–*x*)[0.1BaO–0.4ZnO–0.5B_2_O_3_] with *x* = 1.5 mol % *x*Er_2_O_3_–(100–*x*)[0.1BaO–0.4ZnO–0.5B_2_O_3_] with *x* = 2 mol %	532 nm (5 ns 10 Hz)	0.359 0.476 0.574 0.797	0.902 1.085 1.124 1.431	[10]
20Na_2_O–40Bi_2_O_3_–(40–*x*)B_2_O_3_–*x*Eu_2_O_3_with *x* = 0 mol % 20Na_2_O–40Bi_2_O_3_–(40–*x*)B_2_O_3_–*x*Eu_2_O_3_with *x* = 0.5 mol % 20Na_2_O–40Bi_2_O_3_–(40–*x*)B_2_O_3_–*x*Eu_2_O_3_with *x* = 1 mol % 20Na_2_O–40Bi_2_O_3_–(40–*x*)B_2_O_3_–*x*Eu_2_O_3_with *x* = 1.5 mol % 20Na_2_O–40Bi_2_O_3_–(40–*x*)B_2_O_3_–*x*Eu_2_O_3_with *x* = 1.5 mol %	532 nm (5 ns 1 Hz)	1.55 1.74 1.98 2.16 2.37	1.61 1.94 2.26 2.74 3.28	[15]
10Sb_2_O_3_–20Na_2_O–(70–*x*)B_2_O_3_–*x*Eu_2_O_3_with *x* = 0 mol % 10Sb_2_O_3_–20Na_2_O–(70–*x*)B_2_O_3_–*x*Eu_2_O_3_with *x* = 0.5 mol % 10Sb_2_O_3_–20Na_2_O–(70–*x*)B_2_O_3_–*x*Eu_2_O_3_with *x* = 1 mol % 10Sb_2_O_3_–20Na_2_O–(70–*x*)B_2_O_3_–*x*Eu_2_O_3_with *x* = 1.5 mol % 10Sb_2_O_3_–20Na_2_O–(70–*x*)B_2_O_3_–*x*Eu_2_O_3_with *x* = 2 mol %	532 nm (5 ns 1 Hz)	0.921 1.132 1.532 2.141 2.824	0.426 1.491 2.344 2.712 2.915	[37]

## Data Availability

All the data have been reported in the manuscript.
